# The Changes of HIF-1α and VEGF Expression After TACE in Patients With Hepatocellular Carcinoma

**DOI:** 10.14740/jocmr2496w

**Published:** 2016-02-27

**Authors:** Kang Liu, Xu-Li Min, Juan Peng, Ke Yang, Lin Yang, Xiao-Ming Zhang

**Affiliations:** a Department of Pain Management, Xianyang Hospital, Yan’an University, Xianyang, Shanxi 712000, China; b Department of Radiology, Affiliated Hospital of North Sichuan Medical College, Nanchong, Sichuan 637000, China

**Keywords:** Hepatocellular carcinoma, Transarterial chemoembolization, Vascular endothelial growth factor, Hypoxia-inducible factor-1α

## Abstract

As a common malignant tumor, hepatocellular carcinoma (HCC) has a high prevalence and is a serious threat to human health. The surgical resection rate of HCC is low, and the prognosis is poor. Although transarterial chemoembolization (TACE) is the main treatment for HCC patients who are not candidates for surgical resection, it is not considered a curative procedure. For HCC, poor TACE efficacy or TACE failure may be related to tumor angiogenesis of the residual disease. Among the many regulatory factors in tumor angiogenesis, hypoxia-inducible factor-1α (HIF-1α) and vascular endothelial growth factor (VEGF) play vital roles in this process. In this paper, we conducted a review of the dynamic change and relevance of HIF-1α and VEGF levels after TACE of HCC patients.

## Introduction

As a common malignant tumor, hepatocellular carcinoma (HCC) has a high prevalence and is a serious threat to human health [1]. Due to the hidden nature of onset of HCC, most patients are already at the advanced stage at the time of diagnosis and are not candidates for surgical resection, and thus the prognosis is poor [2-4]. During transarterial chemoembolization (TACE), chemotherapy drugs are perfused locally into the tumor; meanwhile, the blood supply to the tumor is blocked, leading to ischemia, hypoxia, and necrosis of tumor tissue [5]. Currently, the widely recognized Barcelona clinic liver cancer (BCLC) staging system recommends TACE as the standard of care for stage B HCC patients [6-8]. Studies have shown that TACE is effective for HCC and significantly improves the patient’s survival [2, 5, 7, 9-12].

For HCC patients, poor TACE therapy or TACE failure may be related to tumor angiogenesis of the residual disease. Among the many regulators of tumor angiogenesis, hypoxia-inducible factor-1α (HIF-1α) and vascular endothelial growth factor (VEGF) play vital roles in the regulation of tumor angiogenesis of the residual disease after TACE of HCC [13-17]. In this paper, we conducted a review of the dynamic change and relevance of HIF-1α and VEGF levels after TACE of HCC patients.

## TACE in HCC

A normal liver has a dual blood supply from the hepatic artery and portal vein; the portal vein provides 70-75% of the blood supply, and the hepatic artery provides 25-30% of the blood supply. In more malignant hepatic nodules, the blood supply originates less from the portal vein and more from the hepatic artery. HCC is mainly supplied by the hepatic artery. Moreover, advanced stages of HCC are correlated with a greater number of arteries in the HCC [18, 19]. Computed tomography (CT) perfusion imaging has shown that HCC exhibits high arterial and low portal vein perfusions, which are valuable features in the diagnosis and differential diagnosis of HCC [20, 21], as well as in the anatomical basis of TACE.

TACE involves both anti-cancer chemotherapy and embolization of tumor feeding arteries. Perfusion of anti-cancer chemotherapy drugs via feeding arteries increases the local drug concentration at the lesions and reduces the side effects of the drugs. Moreover, embolization of feeding arteries blocks the blood supply of the tumor, resulting in ischemia, hypoxia, and necrosis of the tumor tissue. Many factors affect the efficacy of TACE in the treatment of HCC, and tumor angiogenesis is an important factor. Kenji et al and our previous study have shown that TACE is effective for HCCs with a rich blood supply and is less effective for HCCs with a poor blood supply [22, 23]; these tumors require a multi-disciplinary approach for effective treatment.

Effective monitoring of the treatment response of HCC patients to TACE affects the efficacy evaluation and development of the follow-up treatment regimen, including the time and frequency of repeat chemoembolization [24]. Previous World Health Organization (WHO) and response evaluation criteria in solid tumors (RECIST) criteria require measuring the change in tumor volume on imaging tests, which have significant shortcomings and drawbacks because in some HCC cases, the tumor volume may not change significantly, even though TACE or targeted therapy has been very effective (with no surviving tumor cells) [25]. In recent years, the evaluation of tumor viability has drawn more attention. The European Association for the Study of the Liver (EASL)-recommended modified RECIST (mRECIST) criteria have taken into account that treatment may cause tumor necrosis, and the post-treatment surviving tumors are defined as lesions with contrast uptake during the arterial phase [26].

## HCC and Tumor Angiogenesis

The development, progression, and metastasis of solid tumors are closely related to angiogenesis (vessel-dependent). Studies have shown that the up-regulation of HIF-1α expression is one of the predictive factors for a poor prognosis in many tumor patients [27-29]. For HCCs, nutrients are obtained from the host through tumor blood vessels, and cancer cells are transferred to the host through the tumor blood vessels so that cancer cells may grow in other sites or tissues of the body, ultimately leading to tumor metastasis [30]. As key inducing factors of tumor angiogenesis, HIF-lα and VEGF are involved in all stages of the process [30-33].

HIF-1 functions as a master regulator of oxygen homeostasis in most nucleated mammalian cells and is composed of the constitutively expressed HIF-1β subunit and the highly regulated HIF-1α subunit. Subunit β is constitutively expressed. Currently, it is believed that subunit α, which exhibits sequence homology to subunit β, is the functional subunit that regulates the activation of HIF-1. The expression and transcriptional activity of the HIF-lα protein are subject to precise regulation of the intracellular oxygen level, which occurs on multiple levels, including mRNA expression, protein expression, nuclear localization, and transcriptional activation. Hypoxia stimulates HIF signaling pathways and, in particular, activates HIF-1α [31], which plays a synergistic role in the expression of downstream genes (such as VEGF, p53, and erythropoietin (EPO)) and counteracts anti-proliferative and pro-apoptotic genes (such as NIX, BNIP3, and IGFBP3), thus significantly promoting tumor growth [34, 35].

Ferrara and Henzel [36] first isolated and purified VEGF from the supernatant of bovine pituitary follicular cell culture and named the protein. VEGF is a homodimer glycoprotein that consists of two single chains linked by disulfide bonds, and based on its structure, it is a member of the cystine knot growth factor superfamily. The genes that encode VEGF are located on the short arm of chromosome 6. There are several VEGF splicing isoforms. Among these isoforms, VEGF121, VEGF165 and VEGF189 are the most prominent in human cells. VEGF is one of the most potent angiogenic growth factors. It is highly expressed in HCC patients, induces tumor angiogenesis, and promotes tumor growth and metastasis. VEGF expression is stable and low in normal liver tissue [37, 38]. HIF-1α binds to the promoter region of VEGF and induces transcription and expression of VEGF, resulting in neovascularization and an increased oxygen supply in the tumor tissue [39].

## Changes and Relevance of Serum HIF-1α and VEGF Levels After TACE of HCC Patients

TACE of HCC causes local hypoxia in the tumor, resulting in a series of adaptive changes in the transcription and expression of hypoxia response genes in tumor cells. Hypoxia response genes are mainly regulated by HIF-1α, which induces VEGF expression and promotes neovascularization [15, 40-42]. Due to structural and functional defects, newly formed tumor blood vessels further aggravate hypoxia, and thereby form a vicious cycle leading to tumor recurrence and metastasis.

Studies have shown that dynamic changes in serum HIF-1α and VEGF levels occur after TACE of HCC patients [41-46]. Jia et al [41] investigated the expression levels of serum HIF-1α and VEGF pre- and post-TACE in patients with HCC, and correlations between prognosis factors and serum HIF-1α as well as VEGF levels. Forty consecutive patients with HCC undergoing TACE were enrolled into the study. The study revealed that the expression levels of serum HIF-1α and VEGF in HCC patients were significantly higher than those in control group. One day after TACE, both serum HIF-1α and VEGF levels reached the peak values. One week post-TACE, expression levels of them were decreased, but still significantly higher than those before TACE. The levels of both HIF-1α and VEGF in CR group 1 month post-TACE were significantly lower than those in PR + SD + PD group. Li et al [42] investigated the changes of VEGF level in the course of TACE in 45 HCC patients before, 1, 3, and 7 days after, and 1 month after TACE. The result showed that the VEGF levels increased significantly on the first day after TACE, and then decreased gradually on the third and seventh day post-TACE, but showing no statistical difference with pre-TACE VEGF levels. Ranieri et al [47-51] showed similar results.

However, a few reports revealed that VEGF level increased more slowly after TACE administration [52, 53]. Suzuki et al [52] examined the level of VEGF, AST, ALT and LDH in sera of patients with HCC who underwent transcatheter arterial embolization (TAE) during the course of the treatment. Thirty-eight patients with HCC were studied. Peripheral blood samples were taken before and 1, 3 and 7 days after TAE. Although the level of AST, ALT and LDH reached the peak value within 1 day after TAE, VEGF level increased significantly 7 days later. Chao et al [53] investigated the serum IL-6 and VEGF level before and 1, 3, 5, 7, and 14 days after TACE in 41 patients with HCC. The result showed that the level of serum IL-6 increased rapidly and peaked on day 1 after TACE administration, whereas the level of VEGF increased more slowly and peaked on day 14 after TACE administration.

## Changes and Relevance of HIF-1α and VEGF Protein Expression in Tumor Tissues After TACE of HCC Patients

Currently, there are a few reports of the changes in HIF-1α and VEGF protein expression in tumor tissues after TACE of HCC patients. Xu et al [54] investigated the prognosis and expression of genes regulated by HIF-1α in HCC patients who received preoperative TACE for the first time. The study revealed that HIF-1α protein levels were significantly increased in TACE tissues. In addition, several other studies have reported that TACE stimulates tumor angiogenesis by up-regulating the expression of the VEGF protein [55-59]. Xiao et al [55] performed immunohistochemical staining of specimens from 79 HCC patients who underwent surgical resection after TACE and 57 HCC patients who underwent surgical resection without TACE to detect the changes in VEGF protein expression. The results showed that the VEGF positive rate was significantly lower in patients treated with surgical resection alone than in patients who underwent TACE before surgical resection.

However, Farris et al [60] reported a different result. In the study, patients with HCCs receiving no locoregional therapy (LRT) (control) were compared with LRT treatment groups with conventional TACE (cTACE) or drug-eluting bead TACE (DEB TACE). Tumoral VEGF was significantly different between groups, with the control group having the highest degree of positivity. The study revealed that LRT leads to decreased tumoral VEGF.

In recent years, some researchers have conducted animal experiments to investigate the impact of TACE on HIF-1α and VEGF protein expression. Virmani et al [61] tested the hypothesis that TAE induces expression of HIF-1α within the same rabbit VX2 liver tumor. In their study, seven VX2 tumors were grown in the livers of five New Zealand white rabbits. Pre- and post-TAE tumor biopsy specimens along with post-TAE whole liver tumor sections were stained with an HIF-1α antibody and analyzed to determine the percentage of HIF-1α positive nuclei using a spectral unmixing system mounted on a high-powered microscope. The results indicated that TAE of liver tumors resulted in a statistically significant increase in the mean percentage of HIF-1α expression. The study revealed that hypoxia caused by TAE of VX2 liver tumors activates HIF-1α, a transcription factor that regulates other pro-angiogenic factors. Zhou et al [14, 62, 63] also reported similar results.

## Conclusion

As potent factors of tumor angiogenesis, HIF-1α and VEGF play important roles in the development, progression, and metastasis of HCC and are important for the efficacy evaluation of TACE of HCC patients and the development of individualized treatment regimens. The inclusion of monitoring of the changes in serum HIF-1α and VEGF levels or HIF-1α and VEGF protein expression in tumor tissues after TACE of HCC patients as part of the efficacy evaluation criteria has enhanced the evaluation of HCC treatment. In addition to these methods, in-depth studies that also adopt functional imaging techniques will help to improve the response evaluation criteria in solid tumors [64-70]. Furthermore, further researches are needed to investigate the dynamic changes in HIF-1α expression after TACE of HCC patients and the roles of HIF-1α in the mechanisms of recurrence and metastasis of HCC after TACE.
